# Current trials in erythropoietic protoporphyria: are placebo controls ethical?

**DOI:** 10.1186/s13023-023-02941-w

**Published:** 2023-10-16

**Authors:** Jasmin Barman-Aksözen, Mattia Andreoletti, Alessandro Blasimme

**Affiliations:** 1 International Porphyria Patient Network, Hegarstrasse 3, 8032 Zurich, Switzerland; 2 Department of Health Sciences and Technology, ETH Zurich, Hottingerstrasse 10, 8092 Zurich, Switzerland

## Abstract

A new active substance called “dersimelagon” (MT-7117) is being tested as an alternative treatment option for Erythropoietic protoporphyria (EPP). At the moment, dersimelagon is being tested both in the US and in Europe in a phase III placebo-controlled RCT. However, given the availability of an already approved treatment option for EPP the use of a placebo arm is questionable from an ethics point of view. We analyze the issue and suggest that a noninferiority active-control trial without placebo is an ethically and scientifically more valid design to test the efficacy of dersimelagon as well as other EPP treatments.

## Main text

Erythropoietic protoporphyria (EPP, prevalence 1:100 000) is an inborn error of the heme biosynthesis characterized by phototoxic burn injuries of the endothelial cells lining the blood vessels. The phototoxicity develops within minutes of exposure to visible light and the associated severe neuropathic pain is not responsive to pain medication, including opioids. As the visible light is causing the symptoms, UV (ultra violet)-protective measures alone, like sunscreens have no preventive effects. Patients with EPP, already in their early childhood, develop an ingrained anxiety to be exposed to light, leading to social isolation, depression, and overall impairments in their quality of life and educational and occupational opportunities [1].

In 2014, the European Medicines Agency (EMA) recommended “afamelanotide” (commercial name: Scenesse®) for approval under exceptional circumstances as the first treatment for the prevention of phototoxicity in patients with EPP. The pivotal placebo-controlled randomized clinical trial (RCT) testing afamelanotide showed statistically significant results for its primary endpoint, that is, time in sunlight without pain [2]. However, the EMA in their European Public Assessment Report outlined that it would be contrary to medical ethics principles to collect further evidence of clinical efficacy of afamelanotide in placebo-controlled trials as this would expose patients in the placebo arm to the risk of severe phototoxicity and pain [3]. In the United States, the Food and Drug Administration has granted marketing authorization to afamelanotide in 2019, followed by the approval by the Therapeutic Goods Administration in Australia in 2020.

Currently, a new active substance called “dersimelagon” (MT-7117) is being tested as an alternative treatment option for EPP. Dersimelagon is comparable to afamelanotide as to its mode of action (both are melanocortin receptor agonists), but dersimelagon has a different administration mode – being a tablet instead of a bimonthly slow-release subcutaneous implant formulation.

Dersimelagon has been tested both in the US and in Europe in a phase III placebo-controlled RCT. However, given the availability of the approved treatment option with afamelanotide the use of a placebo arm was questionable from an ethics point of view.

## Placebo controls

Critics argue that placebo-controlled studies are unethical if an effective therapy is available for the condition that affects trial participants. Noninferiority trials employing active controls, so the argument goes, still provide sufficient information about efficacy and should thus be preferred. We believe, however, that placebo-controlled trials are valuable and not always unethical, even when an approved therapy exists, provided the trial design is scientifically sound, participants are duly informed, and they are not wronged or seriously harmed [4]. Guidelines and regulations seem to embrace such middle-ground position too.

The Declaration of Helsinki, for instance, states that “[t]he benefits, risks, burdens and effectiveness of a new intervention must be tested against those of the best proven intervention(s) [unless] for compelling and scientifically sound methodological reasons” the use of placebo controls “is necessary to determine the efficacy or safety of an intervention” and participants in the placebo arm “will not be subject to additional risks of serious or irreversible harm as a result of not receiving the best proven intervention” [5].

Similarly, the Code of Federal Regulations states that an active-treatment study can be considered as an adequate and well-controlled study “where the condition treated is such that the administration of placebo or no treatment would be contrary to the interest of the patients” [6].

Moreover, as a general methodological rule, active-control trials should be undertaken only when – at a minimum – the approved therapy used in the control group has been previously tested against a placebo control. Only in this case can one assume that the control drug had an effect on participants and the trial is informative as to the efficacy of the new drug. Of course, other considerations about the quality of the prior placebo-controlled trials matter to the choice of comparators.

Such considerations cast ethical doubts as to the use of placebo controls in ongoing dersimelagon trials.

## Prodromal endpoints

Recognizing the seriously harmful consequences of daylight exposure for EPP patients, investigators in the dersimelagon trial specified endpoints in terms of minutes of exposure before the appearance of *prodromal symptoms*. In this way, participants in the placebo arm are supposedly protected against the risk of severe phototoxic burns.

Prodromal symptoms like tingling and itching are generally mild and revert quickly. If exposure to sunlight continues, prodromes develop into full-fledged phototoxic burns. Time of exposure until prodromes is thus suggested as a more practicable and acceptable measure to test the efficacy of drugs protecting against the symptoms of EPP, vis-à-vis time in sunlight without pain [7]. However, it is not always possible to immediately retreat from the sunlight. Symptoms may start in the middle of a patient’s way to work, in which case she may not have a choice to retreat, or she might not have a place to hide. In case the patient can`t stop the exposure to light, the tingling and itching rapidly increase in intensity and additional symptoms like the pain appear.

Furthermore, it is disputed whether such early symptoms are to be taken as reversible warning signs or rather the beginning of a phototoxic reaction. The issue, to our knowledge, has not been systematically studied. A stronger evidence-base is needed to support the rationale of using prodromal endpoints in placebo-controlled EPP trials.

For many EPP patients, the areas exposed to light need up to several days to recover from early symptoms. During this time, they are more susceptible to further injury and more sensitive to artificial light, and other factors like heat, cold, pressure or touch (a phenomenon called priming) [1]. Further, the tingling and itching is not always present. At times, the phototoxic reaction directly starts with the pain, or with a combination of milder symptoms and pain. External circumstances such as cold wind or certain light conditions, can determine whether the phototoxic reaction might start with pain or other milder sensations.

Until there is consensus on these matters, it is ethically preferable to minimize the risk of incurring untreatable pain caused by sunlight exposure with the adoption of an active (i.e., afamelanotide) instead of a placebo control.

Further, being melanocortin receptor agonists, afamelanotide and dersimelagon both increase skin pigmentation and cause partial unblinding of the trial participants in the active treatment group – thus undermining the rationale for the use of placebo controls. While this limitation had to be accepted in early trials testing afamelanotide, today it is possible to conduct trials against an approved active comparator which would enhance the quality and scientific robustness of the efficacy measurements. What is more, in case different endpoints are measured, the comparability of the trials results to novel substances is anyway limited.

Other dermatological conditions can be studied through trials employing prodromal endpoints. For instance, prodromal symptoms have shown utility in predicting angio-oedema attacks, or flares in hidradenitis suppurativa (6, 7). Should prodromes be used in clinical studies for such conditions, careful ethical consideration should be given to how patients actually experience such symptoms, so as to avoid unnecessary risks and harm for research participants.

## Conclusion

Striking a reasonable balance between ethics and scientific considerations is of the utmost importance for clinical research, especially in the case of research participants who face serious risk from enrolling in a study, like untreatable severe pain.

Given the availability of an approved treatment against EPP, conducting placebo-controlled trials for novel EPP treatments like dersimelagon and other more recent drug candidates such as “Bitopertin” (DISC-1459) [8], or any future ones, is ethically problematic as it exposes participants to preventable risk of severe harm. In the presence of valid alternatives, minimizing such risks is an ethical imperative. A noninferiority active-control trial without a placebo control is an ethically and scientifically more valid design to test the efficacy of another melanocortin receptor agonist, as well as other EPP treatments.

All stakeholders have a role to play to ensure that such principle is respected.

Regulators and sponsors should be more open to accept evidence from active-control trials especially when the conditions for placebo-controlled ones are not easy to fulfill. Input from Institutional Review Boards and Ethics Review Committees is key in this respect. Such bodies must carefully investigate whether ethically preferable alternatives exist to placebo-controlled trials and promptly communicate their assessment to regulators and sponsors.

To this aim, more research is needed to validate the use of alternative endpoints such as early symptoms of skin-related conditions. Most importantly, it is crucial to meaningfully include patients in deliberations about study design, especially – as we saw with EPP – when endpoints have to do with how individuals experience a condition, its symptoms and its impact on everyday-life activities.

## Data Availability

Does not apply.

## List of Abbreviations

EPP: Erythropoietic protoporphyria (EPP
UV: Ultra violet
EMA: European Medicines Agency
RCT: Randomized clinical trial
